# *PmRunt* regulated by *Pm-miR-183* participates in nacre formation possibly through promoting the expression of *collagen VI-like* and *Nacrein* in pearl oyster *Pinctada martensii*

**DOI:** 10.1371/journal.pone.0178561

**Published:** 2017-06-01

**Authors:** Zhe Zheng, Xiaodong Du, Xinwei Xiong, Yu Jiao, Yuewen Deng, Qingheng Wang, Ronglian Huang

**Affiliations:** 1 Fishery College, Guangdong Ocean University, Zhanjiang, China; 2 Guangdong Technology Research Center for Pearl Aquaculture and Process, Guangdong Ocean University, Zhanjiang, China; Laboratoire de Biologie du Développement de Villefranche-sur-Mer, FRANCE

## Abstract

Heterodimeric PEBP2/CBFs are key regulators in diverse biological processes, such as haematopoietic stem-cell generation, bone formation and cancers. In this work, we cloned runt-like transcriptional factor (designated as *PmRun*t) and CBF β (designated as *PmCBF*) gene, which comprise the heterodimeric transcriptional factor in *Pinctada martensii*. *PmRunt* was identified with an open reading frame that encodes 545 amino acids and has typical Runt domain. Phylogenetic analysis results speculated that runt-like transcriptional factors (RDs) in vertebrates and invertebrates are separated into two branches. In molluscs, PmRunt and other RDs are clustered in one of these branches. Direct interaction between PmRunt and PmCBF was evidenced by yeast two-hybrid assay results. Gene repression by RNA interference decreased the expression level of *PmRunt*, and subsequent observation of the inner surface of the nacre by scanning electron microscopy demonstrated disordered growth. The luciferase activities of reporters that contain promoter regions of *Collagen VI-like* (*PmColVI*) and *PmNacrein* were enhanced by *PmRunt*. Meanwhile, *Pm-miR-18*3 apparently inhibited the relative luciferase activity of reporters containing the 3′-UTR of *PmRunt*. The expression level of *PmRunt* was repressed after Pm-miR-183 was overexpressed in the mantle tissue. Therefore, we proposed that *PmRunt* could be targeted by *Pm-miR-183* and regulate the transcription of *PmColVI* and *PmNacrein* by increasing their transcriptional activity, thereby governing nacre formation.

## Introduction

Polyoma virus enhancer-binding protein 2/core-binding factor (PEBP2/CBF) is a heterodimeric transcription factor composed of two subunits, namely, α and β. In mammals, three genes encode α units that contain conserved DNA binding domains, termed Runt domains (RDs). These runt-related transcription factors (RUNXs) namely, *RUNX1* (AML1/PEBP2αB/CBFA2), *RUNX2* (AML3/PEBP2αA/CBFA1) and *RUNX3* (AML2/PEBP2αC/CBFA3), are involved in different biological processes [1]. *RUNX*s increase their binding affinity to DNA by heterodimerising with core-binding factor subunit beta (CBF β).

RUNX proteins play an essential role in actively cycling cells and regulation of cell proliferation before terminal differentiation. During the development of mammalian skeletal system, RUNX2 is a master regulator of osteoblast differentiation and RUNX1 and RUNX3 regulate cartilage formation [2,3]. RUNX1 and RUNX3 are also required for haematopoietic stem cell generation [4,5]. Notably, regulation of gastrointestinal tract development is an ancient function of RUNX3 [6].

In contrast to vertebrates, invertebrates have no fixed number of RD proteins. *Drosophilia* has four RD genes, whereas *Caenorhabditis elegans*, sea urchin and amphioxus each has a single RD gene [7]. In *C*. *elegans*, RD protein repression can result in apparent malformation of the hypodermis and intestine [8]. The amphioxus RD gene is expressed in endodermal cells adjacent to adult gill bars, which may represent an ancestral form of cartilage [9,10]. However, in sea urchin, a complex expression pattern of runt gene in embryos reflects the multiple roles played by RDs in mammals [11]. However, whether this single-copy RD protein in some species may, to some extent, has multiple functions rather than a certain functional differentiation in mammals is unclear.

Most mollusc species have hard exoskeletons (shells) composed of biocalcified calcium carbonate. In mammals, RD proteins, especially RUNX2, participate in the formation of endoskeletons comprising biocalcified calcium phosphate. These proteins directly or indirectly regulate the expression of components in organic matrix and regulators related to skeletogenesis [12]. In bivalves, one RD protein is identified from scallop and considered to play essential roles in ontogenesis and haemocyte production [13,14]. Meanwhile, whether RD proteins are conserved to regulate biomineralization in shell formation is unknown.

In vertebrates, microRNAs (miRNAs) play critical roles in bone homeostasis, particularly in processes such as regulating the extracellular accumulation, differentiation of osteoblast and osteoclast, secretion of growth factors and expression of transcriptional factors [15]. Several miRNAs are identified in molluscs through Solexa deep sequencing or bioinformatic analysis [16–18]. Some of miRNAs, such as miR-29a and miR-2305, participate in shell formation [19,20], that is, they control biomineralization in low invertebrates and high vertebrates.

In the present study, we identified the RD protein (*PmRunt*) and studied its function related to nacre formation in pearl oysters (*P*. *martensii*). The mechanism by which pm-miR-183 regulate PmRunt was also explored. These findings helped reveal the evolution and functional differentiation of the RD gene family, especially during the regulation of skeleton formation.

## Materials and methods

### Experimental materials

The artificial cultured animal *P*. *martensii* were sampled from the coastal area of Liushawan, Zhanjiang (109°57′E, 20°25′N). The area is located in the South China Sea, which is under the jurisdiction of Guangdong province, China. The sampling process does not involve endangered or protected animals. The pearl oysters used in the experiments had a 5–6 cm shell length and were preconditioned with circulating seawater for 2 days at 25–27°C before use.

### RNA isolation, cDNA synthesis, gene clone and qRT-PCR assay

Total RNA was prepared using Trizol reagent and then treated with DNase I (Promega) to eliminate genomic DNA contamination. Templates for mRNA amplification were prepared using oligo (dT)-adaptor primers and specific primer with M-MLV reverse transcriptase (Promega, USA). Considering the genomic and transcriptome data from our laboratory (data not shown), we obtained partial sequences of *PmRunt* and *PmCBF* in *P*. *martensii*. The 5′ and 3′ ends of *PmRunt* cDNAs were obtained by rapidly amplifying the cDNA ends.

qRT-PCR assay was performed to quantify the expression of target genes. Thermo Scientific DyNAmo Flash SYBR Green qPCR Kit (Thermo Scientific) was used according to the manufacturer’s protocol. Fluorescence was detected in Applied Biosystems 7500/7500 Fast Real-time system (Applied Biosystems, Foster City, CA, USA). The relative expression levels of the target genes were calculated through the 2^−ΔΔct^ method, and U6 or GAPDH were used as reference gene. All PCR primers used in this study are listed in S1 Table.

### Sequence analysis and target gene prediction

Similarity analysis of protein sequence was conducted using the BLAST program from the US National Center for Biotechnology Information (http://blast.ncbi.nlm.nih.gov/Blast.cgi). The deduced amino acid sequence was analysed in Expert Protein Analysis System (http://www.expasy.org). The protein domain was predicted with the simple modular architecture research tool version 5.1 (http://www.smart.emblheidelberg.de/). The multiple alignment of the deduced amino acid sequence of PmRunt or PmCBFβs with other species was performed using the ClustalW multiple alignment program (http://www.ebi.ac.uk/clustalw/). The phylogenetic tree of PmRunt and other species was constructed in Mega 6.0. The sequence accession numbers are listed in S2 Table. Target prediction between *Pm-miR-183* and *PmRunt* was performed using the miRanda and microTar software packages. The same interaction positions were selected as potential target sites. We submitted the PmRunt and CBF sequences, which would be used to construct 3D models, through Phyre2 (http://www.sbg.bio.ic.ac.uk/phyre2/protocol) [21]. Chimera 1.8.1 was used to display these models.

### Plasmid construction

The complete coding sequence of *PmRunt* was subcloned into a pcDNA3.1+ plasmid (purchased from Promega). *PmColVI* and *PmNacrein* promoters were cloned and then inserted into a pGL3-basic reporter vector, respectively (purchased from Promega). The 3′-UTR region of *PmRunt* was subcloned into a pmiR-reporter plasmid (purchased from Ambion). The primers used to construct the plasmids are shown in S1 Table.

### Yeast two-hybrid assay

Yeast two-hybrid assay was performed using Clontech Matchmaker Gold Yeast Two-Hybrid System (Takara, Japan) to detect the possible interactions between PmRunt and PmCBF. In a typical procedure, two sets of gene-specific primers cbf-S/A and Runt-AD-S/A with BamHI–XhoI restriction sites (S1 Table) were designed to amplify the open reading frames (ORFs) of the two genes and fused into pGADT7 and pGBKT7 (Clontech), respectively. PGBKT7-PmCBF and pGADT7-PmRunt plasmids were co-transfected into AH109 and were used as the experimental group. The pGBKT7-53 and pGADT7-T plasmids were used as positive controls, whereas the pGBKT7-Lam and pGADT7-T plasmids were used as negative controls. Both sets of plasmids were co-transfected into AH109. For self-activation detection, the pGADT7-PmRunt and pGBKT7 plasmids and the pGBKT7-PmCBF and pGADT7 plasmids were co-transfected into AH109. Yeasts were grown on plates with double dropout medium (SD/-Leu/-Trp) for 3–5 days at 30°C. The positive monoclonal colony was selected for the five plants above and then spread on the plates with quadruple dropout medium (SD/-Leu/-Trp/-His/-Ade) and quadruple dropout medium supplemented with X-a-Gal (SD/-Leu/-Trp/-His/-Ade/X-a-Gal). The presence of blue cells on the media indicated successful interaction.

### Cell culture transfection assays

Given that no permanent pearl oyster cell line was available, HEK-293T cells were cultured at 37°C in DMEM/HIGE GLUCOSE medium containing 10% fetal bovine serum in a CO_2_ incubator with 5% CO_2_. HEK-293T cells in logarithmic phase were seeded onto a 48-well culture plate, and the number of inoculated cells was 5×10^4^ per hole with 500 μL medium. The density of transfected cells reached 70%–90% on the 2nd day. Plasmid transfection was performed using Lipofectamine^™^ 2000 (Invitrogen) in accordance with the manufacturer’s instructions. The promoter activity of *PmColVI* was tested by transfecting the constructed pGL3-PmColVI vector with the pGL3-basic empty vector as blank control. The pcDNA3.1+ empty vector and pGL3-PmColVI vector were subsequently co-transfected into the HEK-293T cells as negative control. The pcDNA-PmRunt and pGL3-PmColVI vector were then co-transfected into the HEK-293T cells as the experiment group to determine the interactions of PmRunt and promoter region of *PmColVI*. Similarly, pcDNA-PmRunt and pGL3-PmNacrein vectors were co-transfected into the HEK-293T cells as the experiment group to determine the interaction of *PmRunt* and promoter region of *PmNacrein*, with negative control that the pcDNA3.1+ empty vector and pGL3-PmNacrein vector were subsequently co-transfected into the HEK-293T cells. The targeting regulation of *Pm-miR-183* and *PmRunt* was determined by co-transfecting the constructed plasmid pmiR-PmRunt with *Pm-miR-183* or negative control (N.C.). Transfected pmiR-PmRunt was used as blank control. Each cell group must be co-transfected with 4 ng of plasmid pRL-TK vector as internal quality control to determine relative activity in a dual luciferase report system. Luciferase activity was tested using a dual-luciferase assay kit (Promega).

### RNA interference experiment

RNA interference (RNAi) was performed to identify the function of the *PmRunt* gene on the formation of nacre layer in vivo. The primers used to amplify the specific sequence of PmRunt and red fluorescent protein (RFP) are shown in S1 Table. Double-stranded RNA dsRNA was synthesised using the T7 High Efficiency Transcription Kit (TransGen Biotech, JT101) and purified using EasyPure RNA Purification Kit (TransGen Biotech, ER701). The individual injected with 100μL RNase-free water served as blank control. dsRNA-PmRunt or dsRNA-RFP diluted to 30 μg/100 μL with RNase-free water was used in the experimental group or used as negative control. Five individuals of *P*. *martensii* (2 years old; shell lengths measuring between 5 and 6 cm) were used for each treatment. Solution was injected into the adductor muscles of the samples for the first time, respectively. This solution was injected again into the samples after 4 days at the same dose. The mantle pallial tissues of the samples were cut into pieces and immediately stored in liquid nitrogen 8 days after the first injection. The shells were cut, and the inner surface of the shells was observed with a FEIQuanta 200 scanning electron microscope.

### Over-expression of *Pm-miR-183* in vivo

The mimics of *Pm-miR-183* and microRNA N.C. were compounded from Genepharma, Shanghai, China. The sequences are shown in S1 Table. The mimics of Pm-miR-183 and N.C. were diluted in 0.1 μg/μL of RNase-free water, and RNase-free water treatment served as blank control. Five individuals of *P*. *martensii* (2 years old; shell length ranging between 5 and 6 cm) were used for each treatment, and their adductor muscles were injected with 100 μL of the solution, respectively. The mantle pallial of each sample was collected after 72 h.

### Statistical analysis

SPSS software (IBM, Chicago, IL, USA) was used for one-way ANOVA, Duncan’s multiple and *T* test. A difference with *p* of <0.05 was considered statistically significant on the basis of Duncan’s multiple comparison test or *T* test.

## Results

### Cloning and characterization of PmRunt and PmCBF β

**Fig 1:**
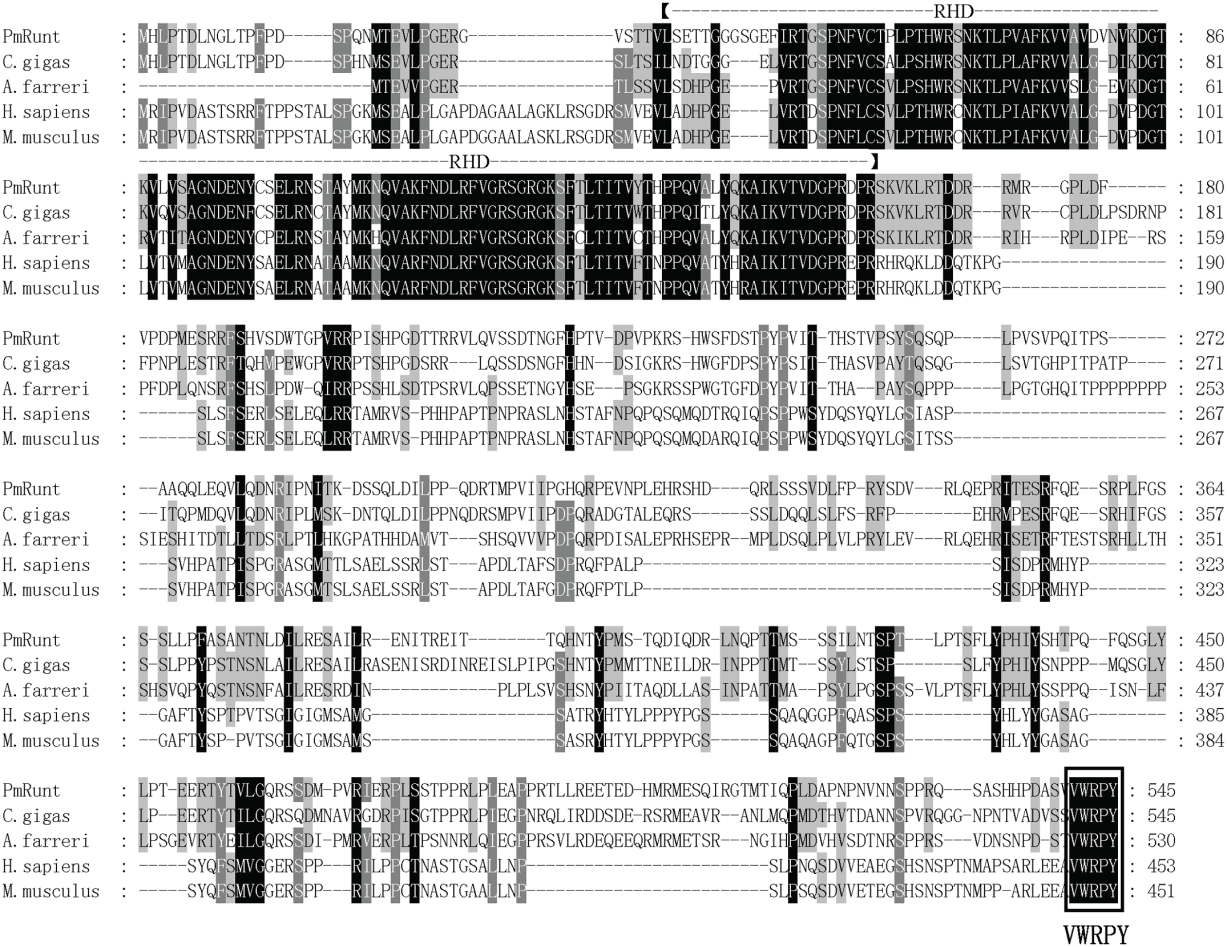
Multiple alignment of PmRunt with other runt-related transcription factors.

**Fig 2:**
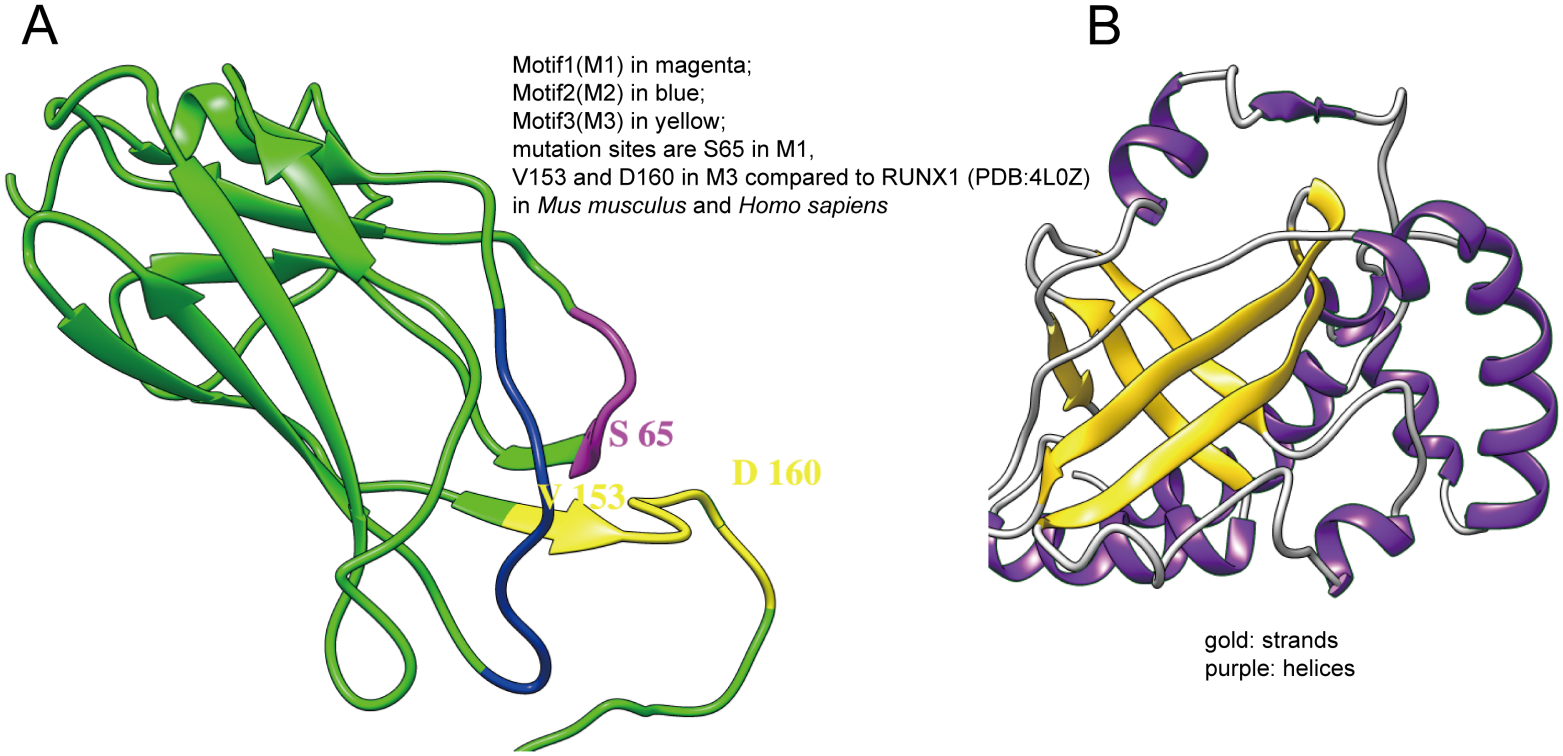
Three-dimensional constructs of PmRunt (A) and PmCBF (B).

The full-length cDNA of *PmRunt* was 2319 bp. The sequences were deposited in GenBank under accession number KY056582. The complete sequence of *PmRunt* contained a 1638-bp ORF encoding a putative protein of 545aa, 5′-untranslated region of 82 bp and 3′-untranslated region of 599 bp (S1A Fig). The predicted molecular weight was 61.19 kDa, and the theoretical isoelectric point was 8.73. The deduced amino acid sequence of PmRunt contained a 129-amino-acid Runt homology domain (RHD; 34–163 aa) in the N-terminal region, which was a DNA-binding domain within the RHD. The multiple sequence alignment of PmRunt with other runt-related transcription factors revealed the conserved RHD and a C-terminal VWRPY motif, with a high identity (51%–77% identity) of RUNX1 in vertebrates (Fig 1). The 3D structure of PmRunt is shown in Fig 2A.

The CBF β, designated as *PmCBF* was identified in *P*. *martensii*. A 591-bp ORF of *PmCBF* encoding a protein of 196 amino acids was obtained (S1B Fig) and had a predicted molecular weight of 23.34 kDa and theoretical isoelectric point of 5.04. The sequences were deposited in GenBank under accession number KY056584. BlastP search indicated that the deduced amino acid sequence of PmCBF showed 58%–82% identity to those of other species from invertebrates to vertebrates. Multiple sequence alignment of PmCBF with other CBFs revealed a conserved CBF β domain (S2 Fig). Moreover, the 3D structures of PmCBF are shown in Fig 2B.

### Phylogenetic relationship analysis

We used maximum likelihood method to construct the phylogenetic tree of RD transcription factors in vertebrates and invertebrates (S3 Fig). The Runt protein from *Amphimedon queenslandica* was selected as the outgroup. Results showed that the RDs in vertebrates and invertebrates could be separated into two branches. Runt from mollusc was clustered into one branch. RUNX1/2/3s in vertebrates could be divided from one common ancestor.

### PmRunt interaction with PmCBF

**Fig 3:**
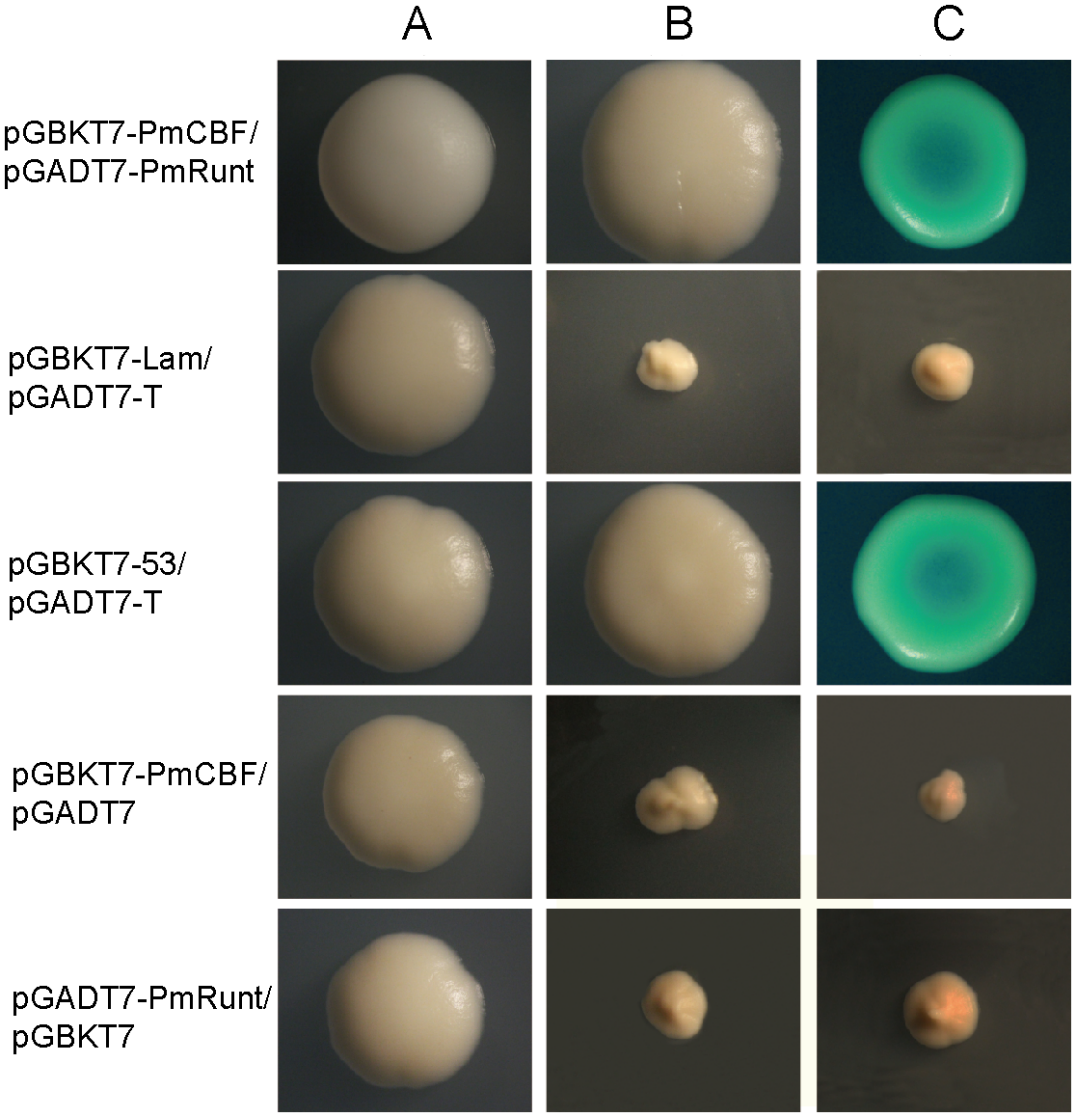
Yeast two-hybrid assay of the interaction between PmRunt and PmCBF. pGBKT7-PmCBF/pGADT7-PmRunt is the experimental group; pGBKT7-Lam/pGADT7-T is the negative control; pGBKT7-53/pGADT7-T is the positive control; pGBKT7-PmCBF/pGADT7, pGADT7-PmRunt/pGBKT7 are the self-activation groups. Line A: Yeasts grow on the plates with double dropout medium (SD/-Leu/-Trp); Line B: Yeasts grow on the plates with quadruple dropout medium (SD/-Leu/-Trp/-His/-Ade); Line C: Yeasts grow on the plates with quadruple dropout medium and supplemented X-a-Gal (SD/-Leu/-Trp/-His/-Ade/X-a-Gal).

In mammals, RUNX proteins need interact with CBFβ to improve affinity to DNA [22]. In this work, we performed yeast two-hybrid assay to investigate the interaction between PmRunt and PmCBF (Fig 3). Five groups of yeasts were grown in an SD/-Leu/-Trp medium. For self-activation detection, pGADT7-PmRunt and pGBKT7, as well as pGBKT7-PmCBF and pGADT7 yeasts, grew in a medium without SD/-Leu/-Trp/-His/-Ade or SD/-Leu/-Trp/-His/-Ade/X-a-Gal, suggesting that PmRunt and PmCBF had no transcriptional activity and did not activate reporter genes. A negative control also did not grow in these media. The yeasts pGBKT7-PmCBF/pGADT7-PmRunt and pGBKT7-53/pGADT7-T1 that grew on plates with SD/-Leu/-Trp/-His/-Ade were white, whereas those that grew on plates with SD/-Leu/-Trp/-His/-Ade/X-a-Gal were blue (Fig 3). These data demonstrated that PmRunt could directly interact with PmCBF.

### PmRunt interference disrupted shell biomineralization

**Fig 4:**
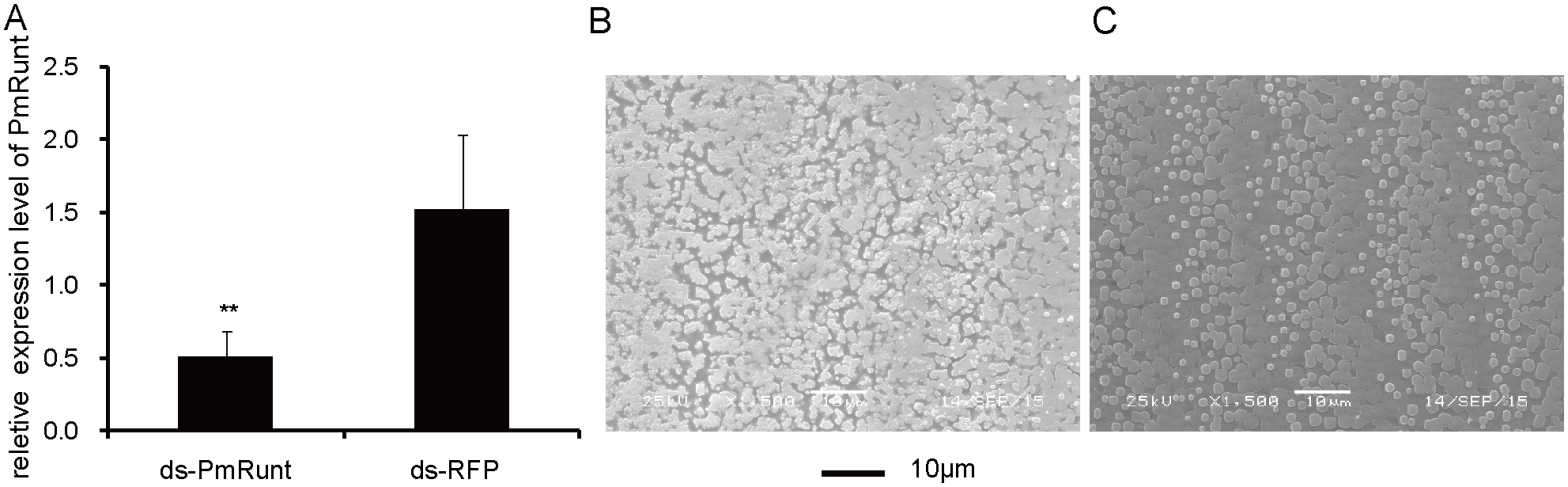
RNA interference and function analysis of PmRunt. Relative expression levels of PmRunt were detected by qRT-PCR in mantle pallials (A) after injection with ds-PmRunt and ds-RFP and then calculated by 2^−ΔΔct^ method. GAPDH was used as the internal reference gene, and the group injected with DEPC-water was the blank control group. ** mean a significant difference (*p* < 0.01). Error bars correspond to mean ± SD. The SEM images of the nacre layer of shell after injection with ds-PmRunt (B) and ds-RFP (C) were detected. The bar represents 10 μm.

To investigate the function of *PmRunt* in shell formation, we used the dsRNA of *PmRunt* to decrease its expression level. Relative to mRNA levels of the negative control injected with dsRNA-RFP, the mRNA levels of *PmRunt* significantly decreased by approximately three-fold in the mantle pallium (*p* < 0.05; Fig 4A). The inner surface microstructure of the shell nacre layer also exhibited disordered growth under SEM (Fig 4B). These results indicated that *PmRunt* played a potential role in nacre-layer formation.

### The transcriptional activities of *PmColVI* and *PmNacrein* could be improved by *PmRunt*

**Fig 5:**
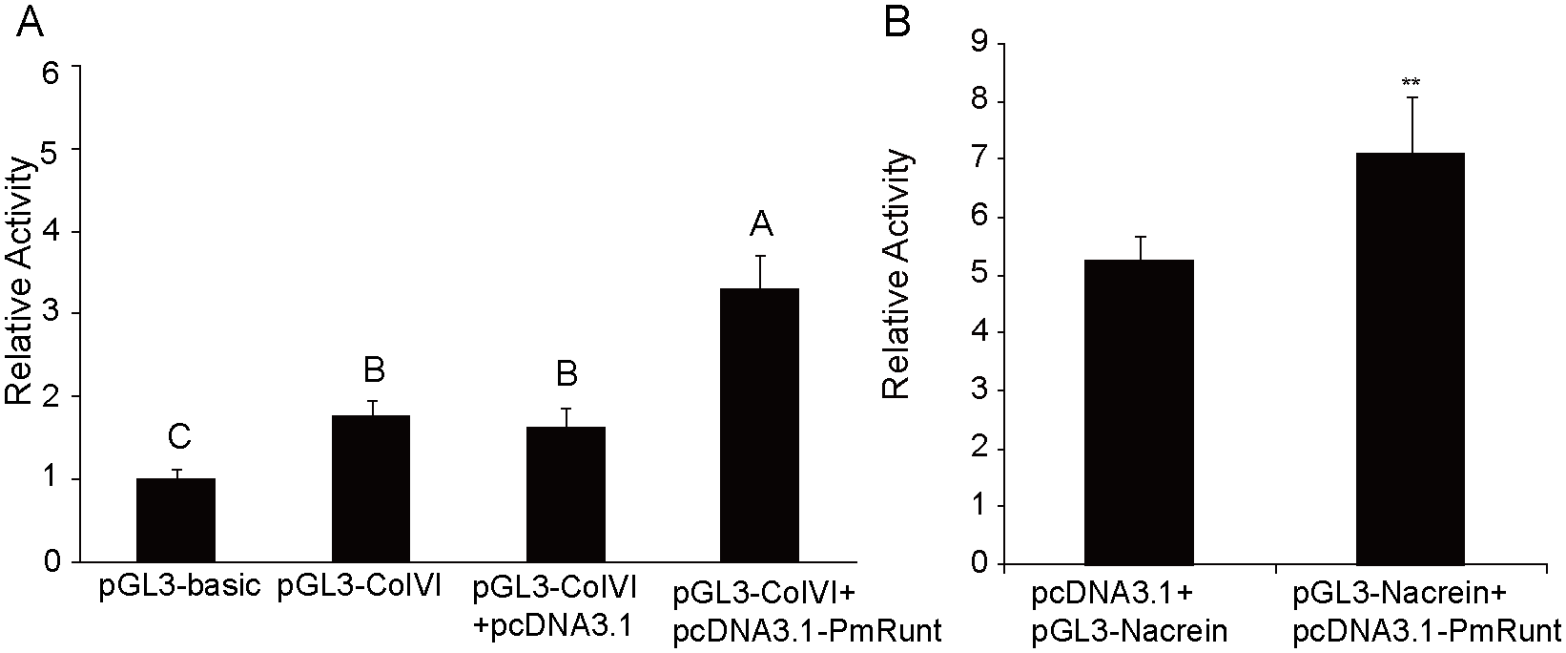
Luciferase reporter assay to assess the ability of PmRunt to activate transcription of the *PmColVI* (A) and *PmNacrein* (B) promoter. The pGL3 basic vector was used as a blank control. The results were analysed by *T* test. Different letters represent significant difference (*p* < 0.01); error bars correspond to mean ± SD.

To validate the regulation mechanism of PmRunt in nacre-layer formation, we tested the interaction of *PmRunt* with *PmColVI* and *PmNacrein* genes [23]. The promoter sequence of *PmColVI* and *PmNacrein* was cloned and constructed into the pGL3-basic reporter. The ORF region of *PmRunt* was inserted into pcDNA3.1+ reporter. The promoter activity of *PmColVI* was a weak. In particular, luciferase activity only increased by 1.8-fold relative to that of the blank control (Fig 5A). Given that the promoter activity of PmNacrein was tested by Sun [24], we directly used the promoter region to construct the reporter vector. After co-transfection with pcDNA-PmRunt in HEK293T cells, the luciferase activities of pGL3-ColVI and pGL3-Nacrein were both significantly up-regulated by ~2- or ~1.3-fold (*p* < 0.01; Fig 5A and 5B), suggesting that PmRunt could slightly enhance the transcriptional activity of *PmColVI* and *PmNacrein* in vivo.

### The expression of *PmRunt* negatively regulated by Pm-miR-183

**Fig 6:**
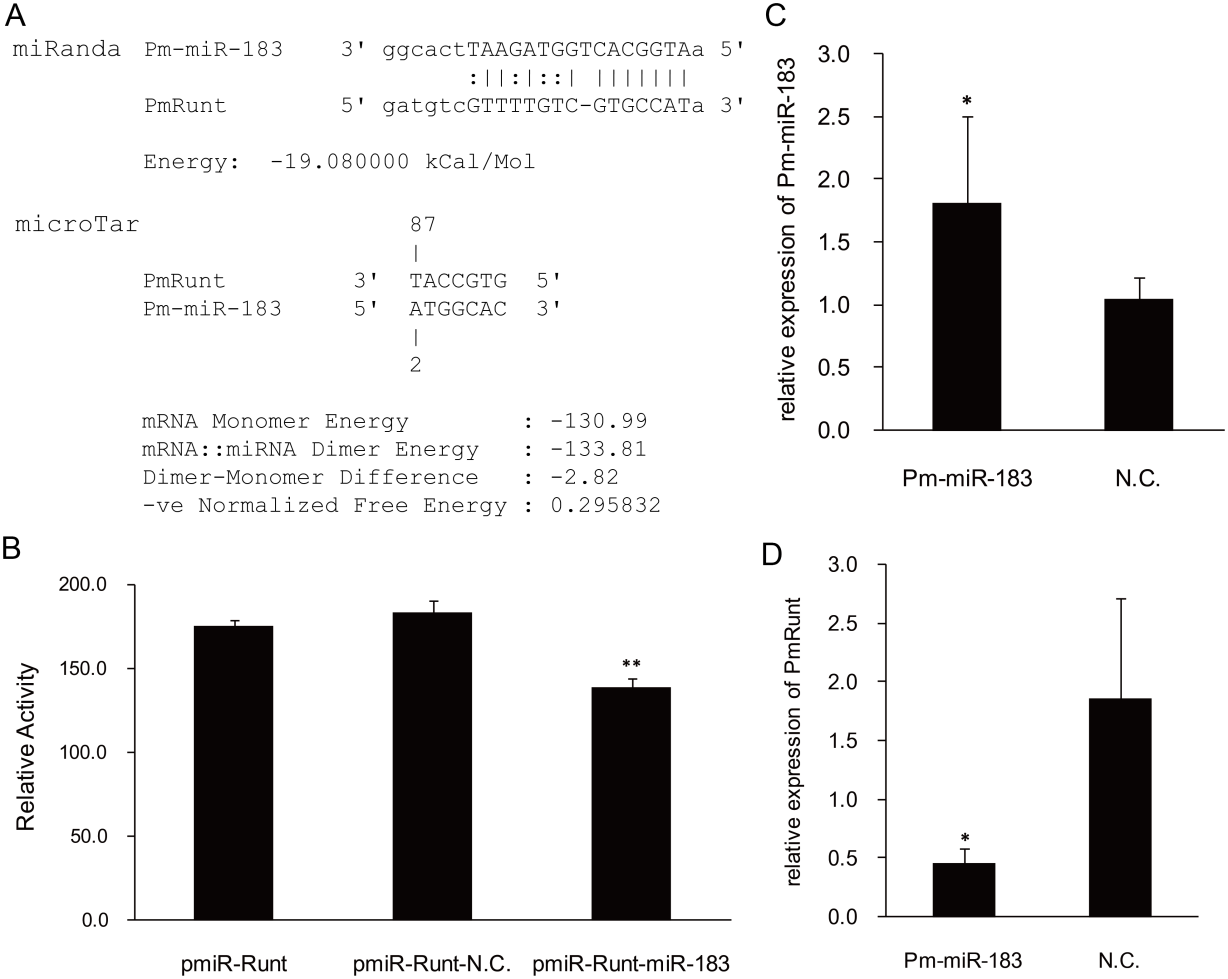
Target verification between Pm-miR-183 and PmRunt. (A) Results of the target prediction between Pm-miR-183 and PmRunt. These results were obtained through the miRanda and microTar software packages. (B) Pm-miR-183 mimics apparently inhibited the luciferase activity of the 3′ UTR of PmRunt. ** represents significant difference (*p* < 0.01). Relative expression of Pm-miR-183 and PmRunt were detected by qRT-PCR after the injection of Pm-miR-183 mimics and N.C. and then calculated through the 2^−ΔΔct^ method. GAPDH or U6 was used as the internal reference gene, and the group injected with DEPC-water was used as blank control group. * represents significant difference (*p* < 0.05). Error bars correspond to mean ± SD.

In a previous study, we identified and characterised miRNAs from pearl oyster *P*. *martensii* through Solexa deep sequencing [16]. For the investigation of the regulation mechanism, miRanda and microTar software were used to predict the potential interactions among miRNAs and PmRunt. The same interaction position between *Pm-miR-183* and 3′-UTR of PmRunt was found (Fig 6A). A DNA fragment with target sites was inserted into the pmiR-reporter vector to confirm the direct interaction. *Pm-miR-183* mimics or negative control mimics were then transfected into HEK293T cells. Results showed that the *Pm-miR-183* mimics decreased the relative luciferase activities by approximately 30% (Fig 6B). The in vivo over-expression of Pm-miR-183 by mimics was observed, with a significant up-regulation of *Pm-miR-183* in the pallial mantle (*p* < 0.05). The expression level of *PmRunt* apparently decreased by approximately 70% relative to that of the negative control (*p* < 0.05). This result is consistent with the luciferase experiment (Fig 6C).

## Discussion

RD proteins widely exist and are present in low sponge species and high mammals. RD proteins can be heterodimerised with CBFs and control diverse biological processes, including embryonic development, osteogenesis, haematopoietic differentiation and cancers [25–28]. In this study, we cloned PmRunt in pearl oyster containing a typical RD and a conserved ‘VWRPY’ in the C-terminal. PmRunt is the highest homologue with Cg-Runt in *Crassostrea gigas*, the homologue gene with RUNX1 in mammal. From the high structure prediction results, we found that PmRunt contains three conserved DNA-binding motifs, namely, ‘RSNKT’, ‘RFVGRSGRG’ and ‘VTVDGPRDPR’. Among those motifs, the following three mutation sites were identified: S in ‘RSNKT’ and V and D in ‘VTVDGPRDPR’, compared with the mammal species C in ‘RCNKT’ and I and E in ‘ITVDGPREPR’ [22,29]. To date, no study has reported that these mutation sites interfere with DNA binding. Moreover, the structure of PmCBF indicated a mixed α/β structure consisting of a partly open five- to six-stranded β-barrel with α-helices packed similarly to CBF β in humans [22]. The directed interaction of PmRunt and PmCBF detected by yeast two-hybrid system also supported the conserved structure

We constructed the phylogenetic tree of RD transcription factors in vertebrates and invertebrates. Runt protein from *A*. *queenslandica* was selected as the outgroup. We found that the RDs in vertebrates and invertebrates were mainly separated into two branches, suggesting independent evolution. The runts from the mollusc species were also clustered into one branch belonging to invertebrates. And RUNX1/2/3s in vertebrates could diverge from a single ancestor. RUNX1 and RUNX2 could be separated from a last common ancestor. A RUNX protein with a single runt in its genome, such as that in mollusc, was identical to RUNX1 in mammals. Robertson [30] proposed that only one *RUNX* gene exists in the lineage between the last common ancestor of metazoan and lophotrochozoan–ecdysozoan. Thus, the branch of RUNX1s in vertebrates may be the ancestor type.

CfRunt in scallop reportedly regulates haemocyte production after bacteria challenge, suggesting similar functions to mammalian RUNX1 [14]. The research of Song [31] also found that the Cg-Runt in *C*. *gigas* exhibited three-bifurcation structures throughout the whole larvae at the later D-veliger and umbo larvae with strong immunoreactivity, suggesting that Cg-Runt is involved in larval haematopoiesis. However, the distinguishable expression of CfRunt in the velum of D-shaped veligar not only indicated haematopoiesis but also indicated the regulation of protoconch formation [13]. The result of the study of Song also showed the apparently expression of Cg-Runt in the velum of umbo veliger and was also highly expressed in the mantle tissue [31]. Therefore, we proposed that runt could be involved in shell formation. In mammals, RUNX1 is also expressed in the primordial cartilage and outer dental epithelium and related to cartilage and tooth formation [2]. We examined the RNA interference of PmRunt in adult *P*. *martensii*. The decrease in PmRunt in the pallial mantle in the main tissue for nacre formation caused the disordered growth of nacre layer, indicating that the PmRunt homologous to RUNX1 played essential roles in biomineralization.

Meanwhile, we found that *PmRunt* could slightly improve the transcription activity of *PmNacrein* in vivo. A previous study showed that the NF-κB signalling pathway, which is mainly related to innate immunity, can regulate nacrein expression by interacting with its promoter [24]. After shell damage, haemocytes, which are the mainly immune cells in bivalves, participate in shell formation [32]. The cellular hypothesis asserts that biomineralization may be directed by haemocytes in *Crassostrea virginica* [33]. In addition to shell formation, mantle tissue participates in immune processes [34]. Meanwhile, Arivalagan [35] found matrix proteins in shell containing domains related to immune functions and proposed that the shell matrix proteins provide the shell with mechanical protection and biochemical defence. Furthermore, the assistance of haemocytes to biomineralization and the coupling of dual regulation functions of Runt and NF-κB in immunity and biomineralization in pearl oyster indicated that the regulation processes of haemocyte/mantle immunity and biomineralization of shell may have co-evolved as a means of self-protection against environmental stress.

Most species have exoskeleton or endoskeleton to adapt to their complex living environment. Several homolog matrix proteins that determine the formation of calcification products are observed in both skeleton types [36–38]. Collagen VI is present in bones with strong cross-linked abilities in matrix proteins, and its reduction induces osteoporosis [39]. Our previous works showed that collagen VI-like gene (*PmColVI*) is significantly expressed in the pallial mantle and responsible for nacre-layer formation in shells [23]. However, whether they share the similar mechanism for the activation or depression of hub genes during biomineralization is unclear. RUNX2 centrally controls skeletal development and osteogenesis by regulating the expression of matrix proteins, such as collagen I and X [40,41]. In the present study, we found that PmRunt mRNA expression was repressed in vivo could cause the disordered growth of the nacre layer, and overexpression of *PmRunt* could slightly improve the transcription activity of *PmColVI* in vitro. These findings suggested that PmRunt, similar to RUNX2 in vertebrates, could be crucial to nacre formation in *P*. *martensii*, as further supported by the conserved regulatory mechanism of skeleton formations in calcium carbonate and calcium phosphate biocalcification systems. However, the regulation of *PmColVI* may not be the main effect of *PmRunt* in nacre formation and thus requires further elucidation.

In mammals, three RD proteins, namely, RUNX1, RUNX2 and RUNX3 were determined. However, their gene numbers in invertebrates vary. For example, *Anopheles gambiae* has three RDs and *D*. *melanogaster* and *Aedes aegypti* have four, similar to tunicates. However, *Ciona interstinalis*, *Strongylocentrotus purpuratus*, *C*. *elegans* and *Chlamys farreri* reportedly have only one [14,42,43] *RUNX* gene. In the present study, only one runt protein was found in *P*. *martensii*. Mammalian RUNX proteins exhibited specific functions in some biological processes. For example, RUNX2 is involved in osteogenesis, and RUNX1 in haematopoietic differentiation. The PmRunt that we identified in *P*. *martensii* was closely homologous with RUNX1 in high mammals but played essential roles in the biomineralization of shells similar to RUNX2. Similar to RUNX1, CfRunt in scallop was observed involved in the regulation of haemocyte production after bacteria challenge [14]. Thus, this kind of single-copy runt protein in shellfish may be relatively versatile and have functions similar to those of RUNX1/2/3 in vertebrates.

Several miRNAs, such as miR-302b, miR-133 and miR-106, can regulate RUNX expression [44–46]. In the current study, we found that *Pm-miR-183* could directly interact with the 3′-UTR of PmRunt and negatively regulate PmRunt expression in vitro and in vivo. MiR-183 in mammals increases osteoclastogenesis by repressing heme oxygenase-1 in bone resorption [47]. In F5M2cells, miR-183 inhibits the metastasis of osteosarcoma, the most common primary malignancy of bones, and influences the migration and invasion of osteosarcomas by targeting Ezrin [48,49]. Therefore, we proposed that miR-183 could participate in calcification-related processes by targeting different genes, although further research is required.

In conclusion, we cloned the sequences of *PmRunt* and *PmCBF* from *P*. *martensii* and confirmed that they could directly form heterodimeric PEBP2/CBF. PmRunt repression could cause the disordered growth of nacre layer of shell to promote the expression of *PmColVI* and *PmNacrein*. *Pm-miR-183* was also involved in the negative regulation of *PmRunt*.

## Supporting information

S1 Fig cDNA sequence and deduced amino acid sequence of PmRunt (A)and PmCBF (B). The shading regions represented Runt domain (A) and CBF domain (B), respectively. (TIF)

S2 Fig Multiple alignment of PmCBF with other core-binding factors. (TIF)

S3 Fig Phylogenetic tree of PmRunt and other species. The blue shading represented the vertebrate branch and the purple shading represented the invertebrate branch. The red branch showed the mollusk cluster. And the five-pointed star pointed the position of PmRunt.
(TIF)

S1 Table Sequences of the primers used for cloning, plasmid construction, and qRT-PCR analyses. (DOCX)

S2 Table Proteins and accession numbers used for phylogenetic analysis of Runt related transcription factors in different species. (DOCX)
