# Supplementary figures and images for: PmRunt regulated by Pm-miR-183 participates in nacre formation possibly through promoting the expression of collagen VI-like and Nacrein in pearl oyster Pinctada martensii
Source: PLoS One. 2017 Jun 1;12(6):e0178561. doi: 10.1371/journal.pone.0178561 (PMC5453546; doi:10.1371/journal.pone.0178561)

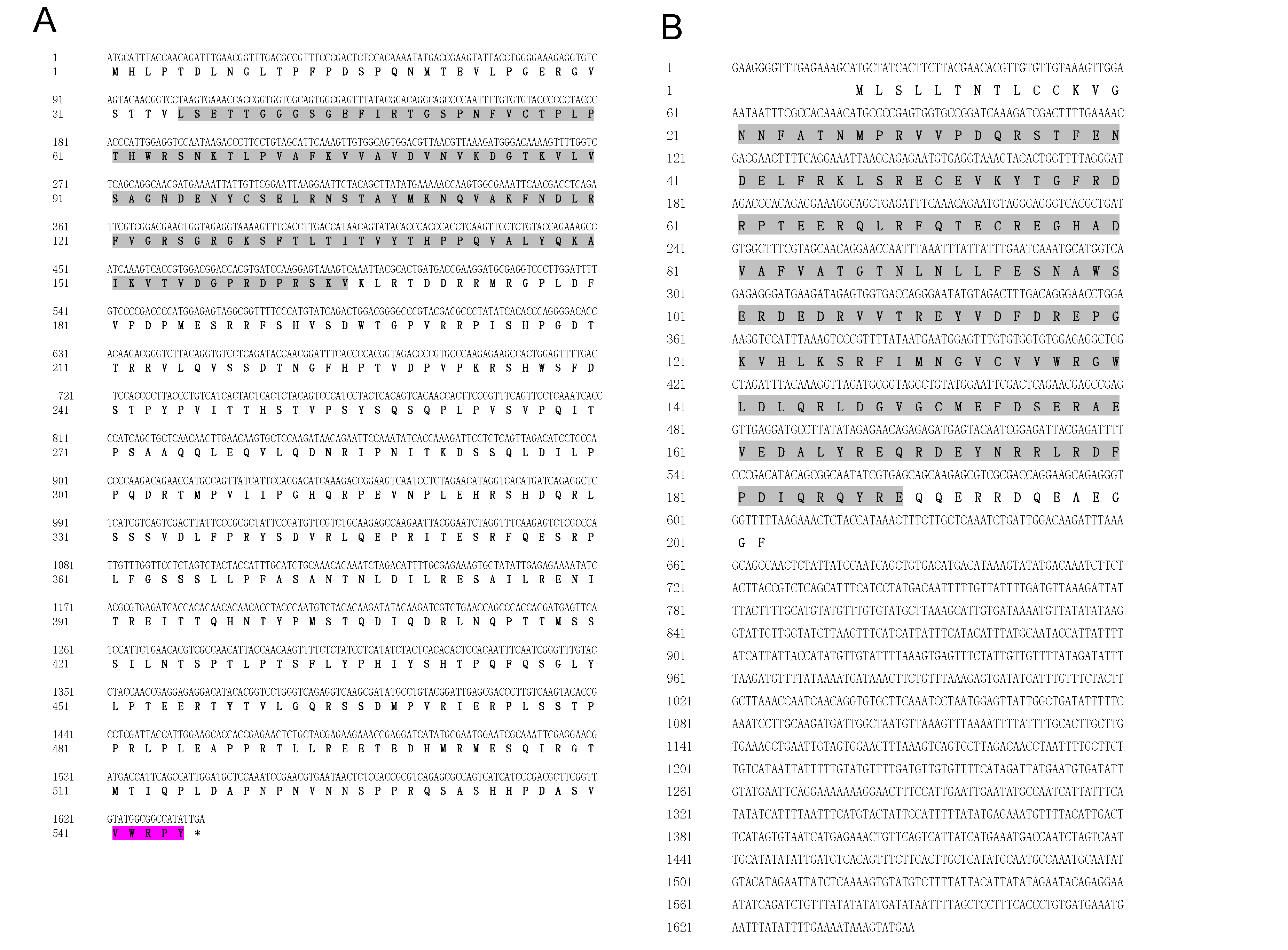

Supplement: S1 Fig — (TIF) [file pone.0178561.s001.tif]

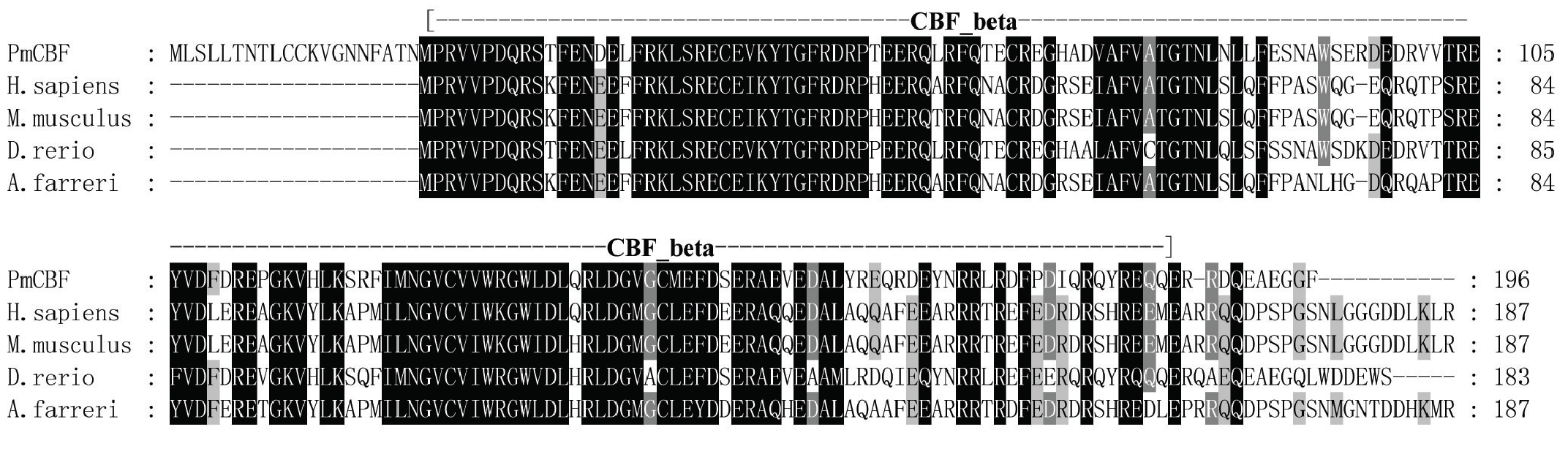

Supplement: S2 Fig — (TIF) [file pone.0178561.s002.tif]

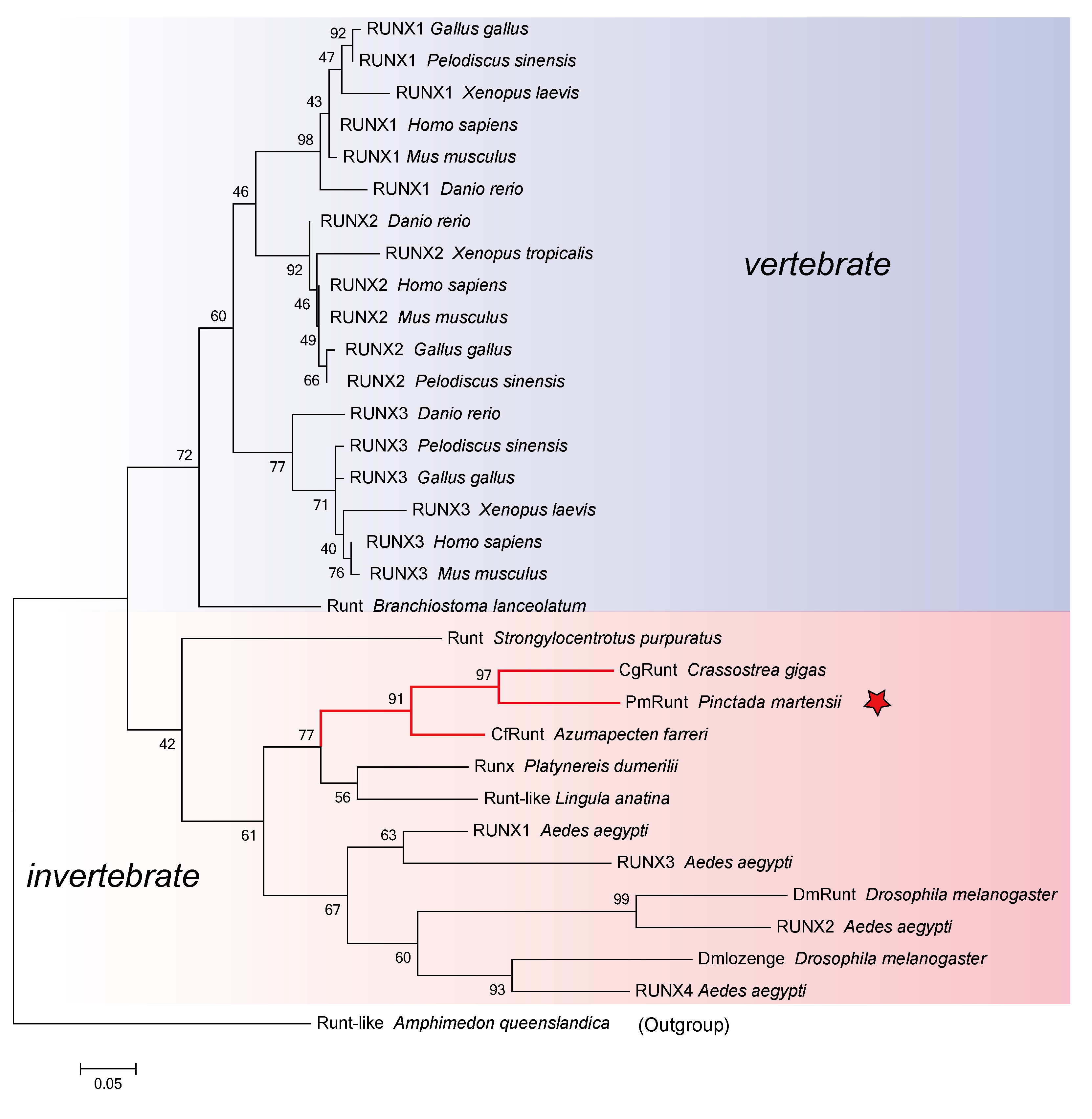

Supplement: S3 Fig — The blue shading represented the vertebrate branch and the purple shading represented the invertebrate branch. The red branch showed the mollusk cluster. And the five-pointed star pointed the position of PmRunt. (TIF) [file pone.0178561.s003.tif]
