# Supplementary material for: PmRunt regulated by Pm-miR-183 participates in nacre formation possibly through promoting the expression of collagen VI-like and Nacrein in pearl oyster Pinctada martensii
Source: PLoS One. 2017 Jun 1;12(6):e0178561. doi: 10.1371/journal.pone.0178561 (PMC5453546; doi:10.1371/journal.pone.0178561)
Supplement: S1 Table — (DOCX) [file pone.0178561.s004.docx]

**S1Table.Sequences of the primers used for cloning, plasmid construction, and qRT-PCR analyses.**

| **Name** | **Sequence** | **Description** |
| --- | --- | --- |
| cbf-S-1 | GAAGGGGTTTGAGAAAGCATGCTAT | fragment |
| cbf-A-1648 | TTCATACTTTATTTTCAAAATATAAATTCATTTCAT | fragment |
| Runt RT(S) | TCCAGGACATCAAAGACCAGAAG | qRT-PCR |
| Runt RT(A) | GGAACCAAACAATGGGCGA | qRT-PCR |
| runt3-outer-1221 | TGTACGGATTGAGCGACCCTTGT | Race |
| runt3-inner-1322 | GGGAATCGCAAATTCGAGGAAC | Race |
| runt3-o-out-1080 | TCTGAACACGTCGCCAACATTAC | Race |
| runt5-inner-35 | GGCGTCAAACCGTTCAAATCTGT | Race |
| runt5-outer-82 | TTTCCCCAGGTAATACTTCGGTCA | Race |
| runt5-o-outer-230 | GCCACAACTTTGAATGCTACAGGA | Race |
| Runt-S | ATGCATTTACCAACAGATTTGAACGG | fragment |
| Runt-A | TCAATATGGCCGCCATACAACC | fragment |
| runt-bamh1 S | CGCGGATCCATGCATTTACCAACAGATTTGAACG | pcDNA3.1+ |
| runt-xhol A | CCGCTCGAGTCAATATGGCCGCCATACAACC | pcDNA3.1+ |
| T7runtsense | TAATACGACTCACTATAGGGTGACCGAAGTATTACCTGGGGA | RNAi |
| T7runtanti | TAATACGACTCACTATAGGGGTCTTGGGGTGGGAGGATGT | RNAi |
| Nacrein-s | CCGCTCGAGTTCTTAGTGTGCTGGTCTTTGT | pGL3-basic |
| Nacrein-a | CCCAAGCTTATGTAATCCTCGTTATCGCA | pGL3-basic |
| U6 reverse | ATTTGCGTGTCATCCTTGC | Reverse transcription |
| U6R | ATTTGCGTGTCATCCTTGC | qRT-PCR |
| U6F | ATTGGAACGATACAGAGAAGATTAG | qRT-PCR |
| miR-183-RT | GTCGTATCCAGTGCGTGTCGTGGAGCTGGCAATTGCACTGGATACGACCCGTGAAT | Reverse transcription |
| miR-183F | AATGGCACTGGTAGAATTCACG | qRT-PCR |
| miR-183R | TGCGTGTCGTGGAGTC | qRT-PCR |
| Runt-pmiR-S | GGACTAGTCCATAGTCGGCAAACATCTGGA | pmiR-Reporter |
| Runt-pmiR-A | CCCAAGCTTGGTATCAAGACTGTGAGCCATTC | pmiR-Reporter |
| GAPDH-S | GCAGATGGTGCCGAGTATGT | qRT-PCR |
| GAPDH-A | CGTTGATTATCTTGGCGAGTG | qRT-PCR |
| cbf-S | CATGCCATGGagATGCTATCACTTCTTACGA | PGBKT7 |
| cbf-A | TGGTTCTGCAGTTAAAAACCACCCTCTGCT | PGBKT7 |
| Runt-AD-S | CGGGATCCatATGCATTTACCAACAGATTTGAACGGTTTG | PGADT7 |
| Runt-AD-A | CCGCTCGAGTCAATATGGCCGCCATACAACCGAAGCGTC | PGADT7 |
| ColVI-S | GGGGTACCCCCTTGGTACAACAAGCAAATCCC | pGL3-basic |
| ColVI-A | CCCTCGAGGGCTGGCACCTTCAAATCAAGTTAG | pGL3-basic |
| Pm-miR-183(sense) | AAUGGCACUGGUAGAAUUCACGG | mimics |
| Pm-miR-183(antisense) | CCGUGAAUUCUACCAGUGCCAUU | mimics |
| N.C.(sense) | UUCUCCGAACGUGUCACGUTT | mimics |
| N.C.(antisense) | ACGUGACCACGUUCGGAGAATT | mimics |
