# Supplementary material for: PmRunt regulated by Pm-miR-183 participates in nacre formation possibly through promoting the expression of collagen VI-like and Nacrein in pearl oyster Pinctada martensii
Source: PLoS One. 2017 Jun 1;12(6):e0178561. doi: 10.1371/journal.pone.0178561 (PMC5453546; doi:10.1371/journal.pone.0178561)
Supplement: S2 Table — (DOCX) [file pone.0178561.s005.docx]

**S2 Table. Proteins and accession numbers used for phylogenetic analysis of Runt related transcription factors in different species**

| **Protein Name** | **Species** | **accession numbers** |
| --- | --- | --- |
| RUNX1 | *Gallus gallus* | NP_990558.1 |
| RUNX1 | *Pelodiscus sinensis* | XP_006126068.2 |
| RUNX1 | *Xenopus laevis* | NP_001079966.1 |
| RUNX1 | *Homo sapiens* | NP_001001890.1 |
| RUNX1 | *Mus musculus* | sp\|Q03347 |
| RUNX1 | *Danio rerio* | NP_571678.1 |
| RUNX2 | *Danio rerio* | NP_998023.1 |
| RUNX2 | *Xenopus tropicalis* | NP_001128588.1 |
| RUNX2 | *Homo sapiens* | *NP_001019801.3* |
| RUNX2 | *Mus musculus* | EDL23422.1 |
| RUNX2 | *Gallus gallus* | NP_989459.1 |
| RUNX2 | *Pelodiscus sinensis* | XP_006120751.2 |
| RUNX3 | *Danio rerio* | NP_571679.2 |
| RUNX3 | *Pelodiscus sinensis* | XP_006130530.1 |
| RUNX3 | *Gallus gallus* | XP_015153219.1 |
| RUNX3 | *Xenopus laevis* | ADE05306.1 |
| RUNX3 | *Homo sapiens* | AAH13362.1 |
| RUNX3 | *Mus musculus* | NP_062706.2 |
| Runt | *Branchiostoma lanceolatum* | AAN08565.1 |
| Runt | *Strongylocentrotus purpuratus* | NP_999779.1 |
| CgRunt | *Crassostrea gigas* | EKC36272.1 |
| CfRunt | *Azumapecten farreri* | AGI44582.1 |
| Runx | *Platynereis dumerilii* | AGS55453.1 |
| Runt-like | *Lingula anatina* | XP_013413919.1 |
| RUNX1 | *Aedes aegypti* | ACF35310.1 |
| RUNX3 | *Aedes aegypti* | ACF35312.1 |
| DmRunt | *Drosophila melanogaster* | AAC27780.1 |
| RUNX2 | *Aedes aegypti* | ACF35311.1 |
| Dmlozenge | *Drosophila melanogaster* | AAF35308.1 |
| RUNX4 | *Aedes aegypti* | ACF35313.1 |
| Runt-like | *Amphimedon queenslandica* | XP_011407123.1 |
| PmRunt | *Pinctada martensii* | KY056582 |
